# Evaluation of the X-linked modifier loci for Leber hereditary optic neuropathy with the G11778A mutation in Chinese

**Published:** 2010-03-11

**Authors:** Yanli Ji, Xiaoyun Jia, Shiqiang Li, Xueshan Xiao, Xiangming Guo, Qingjiong Zhang

**Affiliations:** 1 State Key Laboratory of Ophthalmology, Zhongshan Ophthalmic Center, Sun Yat-sen University, Guangzhou, China; 2 Institute of Clinical Transfusion, Guangzhou Blood Center, Guangzhou, China

## Abstract

**Purpose:**

To test the association of the X-chromosome regions (Xp21.1–q21.2 and Xq25–27.2) with Leber hereditary optic neuropathy (LHON) in Chinese patients.

**Methods:**

One hundred and seventy-five male LHON patients with the G11778A mutation and 100 unrelated normal males participated. Twelve microsatellite markers and four single-nucleotide polymorphisms (SNPs) were genotyped for patients and controls. A χ^2^ or Fisher’s exact test was used to compare the frequencies of genotypes as well as haplotypes in the two groups.

**Results:**

Significant differences between patients and controls were found in two isolated microsatellite markers (DXS6803: χ^2^=37.17, p=2.45×10^−5^; DXS984: χ^2^=33.88, p=1.66×10^−6^) based on genotype frequencies. However, no significant differences for genotype and haplotype frequencies were found in the other 14 markers located in the two reported regions of Xp21.1–q21.2 and Xq25–27.2.

**Conclusions:**

Our results provide suggestive evidence of X-linked modifiers on the expression of LHON. Further studies are needed to identify the exact nuclear genes that might affect LHON expression.

## Introduction

Leber hereditary optic neuropathy (LHON, OMIM 535000) is one of the best studied mitochondrial genetic diseases. The prevalence of LHON is about 1 in 8,500 individuals in the general population of North East England [1]. The majority of LHON cases are caused by three common mitochondrial DNA (mtDNA) mutations, G11778A in the *ND4* gene [2], T14484C in the *ND6* gene [3,4], and G3460A in the *ND1* gene [5,6]. The distribution patterns of these three primary mutations differ remarkably among populations of Europe and East Asia [7,8] and about 90% of LHON cases among Chinese are associated with the G11778A mutation [8].

Only about one third of carriers of the three common mutations will develop LHON, and male carriers have a much higher risk of developing the disease than females. The incomplete penetrance and sex bias of LHON are not well explained by primary mtDNA mutations alone, suggesting that environmental [9-11] or additional genetic factors may contribute to the expression of LHON. Beyond primary mtDNA mutations, other genetic factors that might affect the clinical expression of LHON include additional mtDNA mutations [12], heteroplasmy [13,14], mtDNA haplogroup [7,15-19], and potential nuclear genes such as X-chromosome modified loci [20]. In European families, clear evidence demonstrates that the risk of visual failure is higher when G11778A or T14484C mutations are present in haplogroup J and when G3460A is present in haplogroup K, but is lower when G11778A exists in haplogroup H [7]. The effect of haplogroup J was narrowed to subclades J1c and J2b [19]. Our previous study showed that haplogroup M7b1’2 could increase the risk of visual failure and that M8a might have a protective effect in Chinese families with LHON, which (results of M7b12 and M8a) differ from those found among Europeans [21,22]. However, the effect of mtDNA haplogroups could only partly explain the different penetrance among different families. It could also not explain different penetrance within the same family where all maternal offspring have the same mutation under the same mtDNA background, yet some individuals develop the disease while others do not, and male family members are more likely than females to have the disease.

Previous segregation analysis found that some pedigrees are consistent with an X-linked susceptibility allele [23,24], leading to efforts to map and identify the suspected X-linked modified gene. However, linkage analysis of X-chromosome markers resulted in a series of inconsistent results [25-27]. Recently, Hudson et al. suggested that nuclear modifiers might be more common in the general population than the relatively rare primary mtDNA mutations [28]. Using a nonparametric complex-disease-mapping strategy, they identified an X-chromosomal haplotype DXS8090 (166)/DXS1068 (258) in the Xp21.1–q21.2 region as a risk factor in Europeans, which is independent of the mtDNA background and could well explain the variable penetrance and sex bias in the studied pedigrees. In a recent study, X-chromosomal linkage analysis in a large Brazilian family with the G11778A mutation on a haplogroup J background revealed a novel LHON susceptibility locus on chromosome Xq25–27.2 [29]. Considering the extreme high rate of false-positive results in genetic association studies [30-35], replication is the first priority in a genetic association study of complex traits. In addition, it is necessary to test whether this X-chromosome locus also affects the clinical expression of LHON among Chinese, although we have seen differences in mtDNA haplogroups [7,21] as well as in sex bias (the male to female ratio was 2.2:1 to 2.4:1 among Chinese [8,21] but 3.7:1 to 12.4:1 in Caucasians [36-38]).

Here, we studied the distribution of the microsatellite and SNP markers on the two reported loci and the reported high-risk haplotype [DXS8090 (166)/DXS1068 (258)] in the Xp21.1–q21.2 between Chinese with LHON and normal controls.

## Methods

### Patients

One hundred and seventy-five unrelated male LHON probands with the G11778A mutation were identified from our clinic based on mutational detection of G11778A by allele-specific amplification and single-strand conformational polymorphism analysis as previously described [8,21]. In addition, one hundred unrelated normal males (age, gender, and birth-place matched) participated. Of the 175 LHON patients, 55 had a family history of LHON. All participating individuals were from the central and southeast region of China. Informed consent was obtained from participants before the study, conforming to the tenets of the Declaration of Helsinki and following the Guidance for Sample Collection of Human Genetic Disease (National 863-Plan) by the Ministry of Public Health of China. This study was approved by the Institute Review Board of the Zhongshan Ophthalmic Center. Genomic DNA was prepared from venous leukocytes.

### Genotyping of microsatellite markers

We genotyped twelve microsatellite markers, including seven microsatellite markers (DXS8090, DXS1069, DXS1068, DXS6803, DXS8109, DXS1196, and DXS1222) in the Xp21.1–q21.2 region and five microsatellite markers (DXS8074, DXS1211, DXS984, DXS1205, and DXS1227) in the Xq25–27.2 region. Genotyping primers for DXS1068 and DXS1227 (Table 1) were from Panel 28 of the ABI Linkage Mapping Set v2.5 (Applied Biosystems, Foster City, CA). An M13-tailed primer PCR method [39] was used to genotype the other ten microsatellite markers where a 5′6-FAM labeled M13 probe was used (Table 1). The reaction mixture was composed of 0.5 μl reverse primer (10 μM), 0.125 μl M13-tailed forward primer (10 μM), 0.375 μl 5′6-FAM labeled M13 probe (TaKaRa Biotechnology, Dalian, China; 10 μM), 2 μl Template DNA (40 ng/μl), 0.2 μl rTaq polymerase (5 U/μl), 0.8 μl dNTP (2.5 mM each), and ddH_2_O to a total volume of 10 μl. PCR amplification was performed for the initial denaturation at 94 °C for 8 min, followed by 10 cycles of amplification at 94 °C for 15 s, 55 °C for 15 s, and 72 °C for 30 s, an additional 20 cycles of amplification at 89 °C for 15 s, 55 °C for 15 s, and 72 °C for 30 s, and a final extension at 72 °C for 10 min.

**Table 1:** Primers used to amplify DNA fragments encompassing the twelve microsatellite markers and the four single nucleaotide polymorphisms.

**Name**	**Primer sequence (5′-3′)**	**Length of product (bp)**	**Annealing temperature**
DXS8090	M13 tailed-F-CGTTGTAAAACGACGGCCAGTgggtgaaattccatcacaaa	154–172	55 °C
	R-acaaatgcagatgtacaaaaaata		
rs11266282	F-ccaaagatgaccgtgag	666	60 °C
	F-ctgccaatgttctggatgt		
rs11771	F-tggggttttaggtggtga	350	56 °C
	F-aaatgcaaagggtgatgc		
DXS1069	M13 tailed-F-CGTTGTAAAACGACGGCCAGTagcctaacccacataacagc	254–268	55 °C
	R-agctactatattnaccttggtcttg		
DXS1068	F-cctctaaagcatagggtcca	245–259	55 °C
	R-cccatctgagaacacgctg		
DXS8109	M13 tailed-F-CGTTGTAAAACGACGGCCAGTacaggctcggcttattaggg	229–239	55 °C
	R-5′-ctttcagtgccaggcatagg		
rs6623918	F-5′-tctatttccttactttcccaca	436	58 °C
	R-5′-ggaccctttccgcttgat		
rs5923859	F-5′-tattgttgtaaggtgggc	379	56 °C
	R-5′-cttggcttctgctgatat		
DXS6803	M13 tailed-F-CGTTGTAAAACGACGGCCAGTgaaatgtgctttgacaggaa	110–126	55 °C
	R-5′-caaaaagggacatatgctactt-3′		
DXS1196	M13 tailed-F-CGTTGTAAAACGACGGCCAGTctaaattctcctccaccgtg	209–227	55 °C
	R-tttccagagcagattttcagt		
DXS1222	M13 tailed-F-CGTTGTAAAACGACGGCCAGTgcaaaaatccccagcc	234–240	55 °C
	R-ttcattgccatccagattc		
DXS8074	M13 tailed-F-CGTTGTAAAACGACGGCCAGTataaattagccagaggtgttg	221–231	55 °C
	R-5′-ctaggtgtgtctgtaaaggtagg-3′		
DXS1211	M13 tailed-F-CGTTGTAAAACGACGGCCAGTccctccaatctggcagaa	159–175	55 °C
	R-aagacctgggtttggcct		
DXS984	M13 tailed-F-CGTTGTAAAACGACGGCCAGTtttctgtctgccaagtgttt	154–184	55 °C
	R-tactgngccctactccattc		
DXS1205	M13 tailed-F-CGTTGTAAAACGACGGCCAGTcctacgcatgtggctc	184–202	55 °C
	R-attaatggcttagagtactttttca		
DXS1227	F-agaggtccgagtcttccac	77–99	55 °C
	R-ataagggtttactcccccaa		
M13 probe	CGTTGTAAAACGACGGCCAGT	21	x

Primers used to amplify DNA fragments encompassing the twelve microsatellite markers (DXS8090, DXS1069, DXS1068, DXS8109, DXS6803, DXS1196, DXS1222, DXS8074, DXS1211, DXS984, DXS1205, and DXS1227) and the four SNPs (rs11771, rs11266282, rs5923859, and rs6623918).

Fluorescence-labeled PCR products were separated by capillary electrophoresis using an ABI 3100 genetic analyzer. The lengths of the PCR products were calculated using GeneScan^TM^ 400HD size standards and analyzed using Genemapper software (Applied Biosystems). For the ten microsatellite markers using the M13-tailed primer PCR method, the length of fragments was adjusted (the real length being 21 bp shorter due to the addition of a 21 bp M13-tailed probe on the forward primer).

### Genotyping of single nucleotide polymorphisms

**Figure 1:**
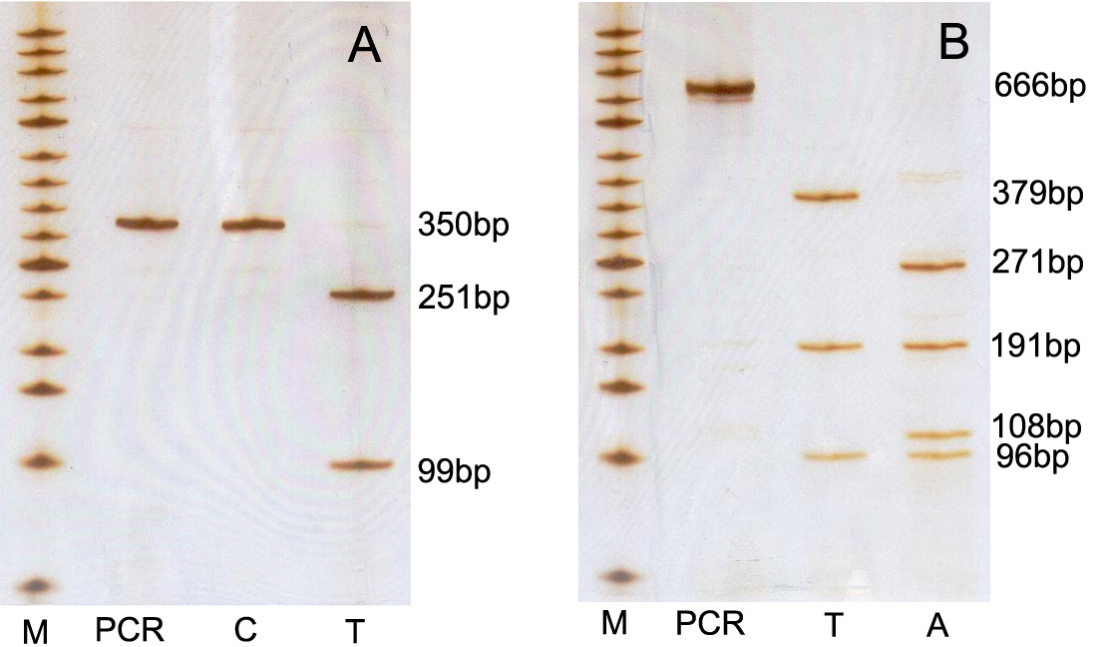
Polymerase chain reaction-restriction fragment length polymorphism (PCR-RFLP) analysis of rs11771 and rs11266282 in LHON patients and normal controls. **A**: The C/T genotype of rs11771 in the DYNLT3 gene was analyzed using HindIII digestion. **B**: The T/A genotype of rs11266282 in the LANCL3 gene was analyzed using HinfI digestion. M: Size marker of 50 bp DNA ladder (from bottom to top: 50 bp, 100 bp, 150 bp, 200 bp, 250 bp, 300 bp, 350 bp, 400 bp, 450 bp, 500 bp, 600 bp, 700 bp, 800 bp, 900 bp, and 1000 bp).

Four SNPs were genotyped. Of the four, rs11771 and rs11266282 in the Xp21.1–q21.2 region were genotyped by polymerase chain reaction (PCR)-restriction fragment length polymorphism analysis, where the amplicons were digested by the restriction endonucleases HindIII and HinfI (TaKaRa Biotechnology), respectively (Table 2). The digested products were separated by 10% PAGE (PAGE; Figure 1). The other two SNPs (rs6623918 and rs5923859) in the Xp21.1–q21.2 region were genotyped by cycle sequencing. The primers used to amplify the fragments harboring these four SNPs are listed in Table 1.

**Table 2:** Enzyme and digestion fragments for RFLP analysis of two SNPs

**SNP**	**Genotype**	**Enzyme**	**Digestion fragments (bp)**
rs11771	C	HindIII	350
	T		251/99
rs11266282	T	HinfI	379/191/96
	A		271/191/108/96

Note: There are only two genotypes of each SNP because two makers lies in X chromosome and two groups of samples are all male.

### Statistical analysis

Distributions of the genotype and haplogroup frequencies of the sixteen markers in the Xp21.1–q21.2 and Xq25–27.2 regions were compared between patients and controls using the chi-square or Fisher’s exact test (SPSS13.0, Chicago, IL). The haplotypes of the two reported markers (DXS8090 and DXS1068) were constructed using PHASE software. A p value of 0.05 or less was regarded as statistically significant, based on previous reports [28].

## Results

**Figure 2:**
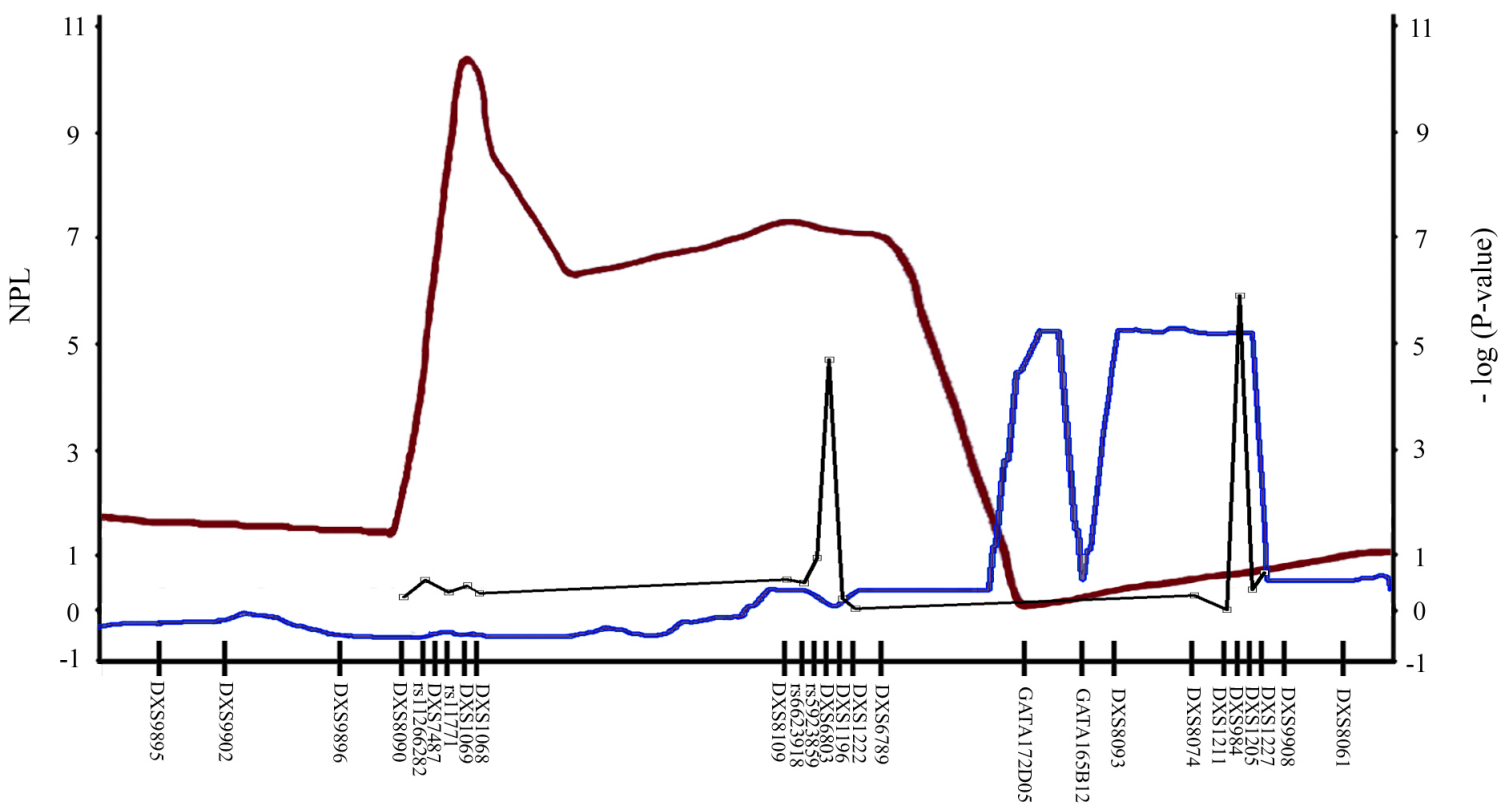
Ideogram of the modifier loci for LHON on the X-chromosome. Auburn and blue lines show the results of Hudson et al. [28] and Shankar et al. [29], respectively, where the nonparametric linkage score (NPL) is listed on the left vertical axis. Black line shows the results of our study, where the –log (p value) on the right vertical axis.

Twelve microsatellite markers and four SNPs were successfully genotyped except for a few samples (which failed to generate amplicons after several attempts). The locations of the analyzed markers on the X-chromosome are shown in Figure 2. The genotyping results for the twelve microsatellite markers are listed in Table 3 and for the four SNPs in Table 4. Two of the sixteen markers yielded significant differences between cases and controls, namely DXS6803 (χ^2^=37.17, p=2.45×10^−5^) and DXS984 (χ^2^=33.88, p=1.66×10^−6^). No statistically significant difference was found in the distribution of genotyping frequencies for the other fourteen markers between LHON patients and controls (Table 3, Figure 2).

**Table 3:** The genotypes distribution of twelve microsatellite markers between the LHON patients and normal controls.

**Genotype**	**Length (bp)**	**LHON (n=175)**	**Normal controls (n=100)**	**χ2 value**	**p value**
DXS8090	152	1	0	7.826	0.55
	154	5	4		
	156	1	1		
	158	10	2		
	160	21	8		
	162	43	32		
	164	74	47		
	166	14	5		
	168	5	1		
	170	1	0		
DXS1069	253	2	1	4.3	0.335
	255	30	24		
	257	137	73		
	257	4	0		
	263	2	2		
DXS1068	251	2	1	6.458	0.472
	253	64	28		
	255	8	3		
	257	21	14		
	259	63	47		
	261	10	4		
	263	0	1		
	265	1	0		
DXS8109	221	1	0	9.247	0.262
	225	0	1		
	227	3	3		
	229	3	0		
	231	13	15		
	233	133	68		
	235	15	9		
	237	5	4		
	241	2	0		
DXS6803	106	2	3	37.174	2.45×10–5
	108	1	0		
	110	28	12		
	112	11	9		
	114	31	11		
	116	44	48		
	118	47	5		
	120	1	0		
	122	10	10		
	126	0	1		
DXS1196	204	1	0	10.26	0.591
	206	2	0		
	208	53	23		
	210	72	46		
	212	12	13		
	214	15	6		
	216	3	2		
	218	1	0		
	220	6	2		
	222	4	3		
	224	2	3		
	226	3	0		
	228	1	1		
DXS1222	227	0	1	3.733	0.897
	229	2	2		
	231	29	16		
	233	102	60		
	235	26	12		
	237	14	9		
	239	1	0		
	241	1	0		
DXS8074	220	143	88	3.393	0.51
	222	3	0		
	224	1	0		
	226	27	11		
	228	1	1		
DXS1211	157	48	32	3.662	0.925
	159	1	0		
	161	45	24		
	163	27	10		
	165	1	1		
	167	7	3		
	169	8	5		
	171	32	22		
	173	6	3		
DXS984	161	1	0	33.879	1.659×10–6
	163	1	1		
	165	40	11		
	167	98	76		
	169	30	2		
	171	0	2		
	173	1	1		
	175	2	6		
	179	2	0		
DXS1205	179	3	1	12.365	0.402
	181	4	3		
	183	18	7		
	185	3	2		
	187	6	4		
	189	28	10		
	191	60	48		
	193	26	7		
	195	2	4		
	197	5	3		
	199	12	7		
	201	2	2		
	203	3	2		
DXS1227	77	1	0	10.058	0.196
	79	4	1		
	83	108	59		
	85	33	15		
	87	2	2		
	89	2	0		
	91	19	21		
	95	6	1		
	97	0	1		

Note: The Fisher' s exact test was used to compare the frequency of the twelve microsatellites between the two groups.

**Table 4:** The genotype distribution of the four SNPs between the LHON patients and controls

**SNP**	**Genotype**				**χ2**	**P**
**LHON (n=175)**		**Controls (n=100)**	
rs11266282	A (117)	T (53)	A (61)	T (37)	1.206	0.272
rs11771	C (133)	T (42)	C (80)	T (20)	0.583	0.445
rs6623918	G (150)	A (25)	G (81)	A (19)	1.052	0.305
rs5923859	A (142)	G (32)	A (89)	G (11)	2.622	0.105

Haplotypes of the reported markers DXS8090/DXS1068 were constructed using PHASE software (Table 5). There was no statistically significant difference in the distributions of these reported haplotypes between LHON patients and controls.

**Table 5:** The distribution of DXS8090-DXS1068 haplotype between the LHON patients and normal controls

**Number**	**DXS8090-DXS1068**	**LHON (n=175)**	**Controls (n=100)**
1	164 251	1 (1%)	1 (1%)
2	154 253	2 (1%)	1 (1%)
3	160 253	8 (5%)	2 (2%)
4	162 253	19 (11%)	14 (14%)
5	164 253	23 (13%)	10 (10%)
6	166 253	6 (3%)	1 (1%)
7	160 255	2 (1%)	1 (1%)
8	162 255	3 (2%)	1 (1%)
9	164 255	2 (1%)	1 (1%)
10	154 257	1 (1%)	1 (1%)
11	160 257	1 (1%)	1 (1%)
12	162 257	8 (5%)	4 (4%)
13	164 257	7 (4%)	7 (7%)
14	158 259	4 (2%)	1 (1%)
15	160 259	6 (3%)	4 (4%)
16	162 259	8 (5%)	10 (10%)
17	164 259	34 (19%)	25 (25%)
18	166 259	6 (3%)	4 (4%)
19	168 259	2 (1%)	1 (1%)
20	162 261	1 (1%)	2 (2%)
21	164 261	5 (3%)	1 (1%)
22	other haplotypes	26 (15%)	7 (7%)

Note: The χ2 value of Fisher's exact test was 12.468 and p value was 0.931.

## Discussion

Several studies have shown that the incomplete penetrance and sex bias of LHON are associated with nuclear modifier genes on the X-chromosome. Recently, DXS8090 (166)/DXS1068 (258) haplotypes in the Xp21.1–q21.2 region were shown to modulate the clinical expression of LHON in European patients [28]. This effect is independent of the mtDNA genetic background and could explain the variable penetrance and sex bias well in these pedigrees. Our results failed to confirm any DXS8090/DXS1068 haplotype with LHON expression among Chinese, but did find a significant difference in a nearby marker (DXS6803: χ^2^=37.17, p=2.45×10^−5^) in the Xp21.1–q21.2 region. This marker is located in the broader linkage region but not in the highly significant fine mapping region reported by Hudson et al. [28]. In addition, our study design of case–control series is different from that of Hudson et al. [28] whose controls were unaffected family members, which may partly explain our discrepant findings. However, a common locus may be detected by either strategy unless it is ethnic-specific.

In a recent study, X-chromosomal linkage analysis in a large Brazilian family with a G11778A mutation on a haplogroup J background revealed a novel LHON susceptibility locus on chromosome Xq25–27.2 [29]. We genotyped five microsatellite markers (DXS8074, DXS1211, DXS984, DXS1205, and DXS1227) in the Xq25–27.2 region. Our results showed that DXS984 differed significantly (χ^2^=33.88, p=1.66×10^−6^) between LHON patients and controls, supporting a possible modifier locus in this region. These results need to be confirmed by additional studies, as two other nearby markers (DXS1211 and DXS1205) did not support the association.

Significant association for isolated markers is not uncommon and has been reported even in a genome-wide association study [40]. Replication and confirmation remains a challenge in association studies. Considering that most genetic risk factors (about 95%) reported for many other complex traits have been false positives [30-33], we must interpret our results with caution at this stage. Further linkage and genome-wide association studies on Chinese families with LHON are essential to provide additional information about the X-linked modifier gene in the Chinese population.

## References

[1] Man PY Griffiths PG Brown DT Howell N Turnbull DM Chinnery PF The epidemiology of Leber hereditary optic neuropathy in the North East of England. Am J Hum Genet 2003 72 333 9 12518276 10.1086/346066 PMC379226

[2] Wallace DC Singh G Lott MT Hodge JA Schurr TG Lezza AM Elsas LJ 2nd Nikoskelainen EK Mitochondrial DNA mutation associated with Leber's hereditary optic neuropathy. Science 1988 242 1427 30 3201231 10.1126/science.3201231

[3] Johns DR Neufeld MJ Park RD An ND-6 mitochondrial DNA mutation associated with Leber hereditary optic neuropathy. Biochem Biophys Res Commun 1992 187 1551 7 1417830 10.1016/0006-291x(92)90479-5

[4] Mackey D Howell N A variant of Leber hereditary optic neuropathy characterized by recovery of vision and by an unusual mitochondrial genetic etiology. Am J Hum Genet 1992 51 1218 28 1463007 PMC1682921

[5] Howell N Bindoff LA McCullough DA Kubacka I Poulton J Mackey D Taylor L Turnbull DM Leber hereditary optic neuropathy: identification of the same mitochondrial ND1 mutation in six pedigrees. Am J Hum Genet 1991 49 939 50 1928099 PMC1683233

[6] Huoponen K Vilkki J Aula P Nikoskelainen EK Savontaus ML A new mtDNA mutation associated with Leber hereditary optic neuroretinopathy. Am J Hum Genet 1991 48 1147 53 1674640 PMC1683111

[7] Hudson G Carelli V Spruijt L Gerards M Mowbray C Achilli A Pyle A Elson J Howell N La Morgia C Valentino ML Huoponen K Savontaus ML Nikoskelainen E Sadun AA Salomao SR Belfort R Jr Griffiths P Man PY de Coo RF Horvath R Zeviani M Smeets HJ Torroni A Chinnery PF Clinical expression of Leber hereditary optic neuropathy is affected by the mitochondrial DNA-haplogroup background. Am J Hum Genet 2007 81 228 33 17668373 10.1086/519394 PMC1950812

[8] Jia X Li S Xiao X Guo X Zhang Q Molecular epidemiology of mtDNA mutations in 903 Chinese families suspected with Leber hereditary optic neuropathy. J Hum Genet 2006 51 851 6 16972023 10.1007/s10038-006-0032-2

[9] Tsao K Aitken PA Johns DR Smoking as an aetiological factor in a pedigree with Leber's hereditary optic neuropathy. Br J Ophthalmol 1999 83 577 81 10216058 10.1136/bjo.83.5.577 PMC1723036

[10] Sadun AA Carelli V Salomao SR Berezovsky A Quiros PA Sadun F DeNegri AM Andrade R Moraes M Passos A Kjaer P Pereira J Valentino ML Schein S Belfort R Extensive investigation of a large Brazilian pedigree of 11778/haplogroup J Leber hereditary optic neuropathy. Am J Ophthalmol 2003 136 231 8 12888043 10.1016/s0002-9394(03)00099-0

[11] Isashiki Y Tabata Y Kamimura K Ohba N Genotypes of aldehyde dehydrogenase and alcohol dehydrogenase polymorphisms in patients with Leber's hereditary optic neuropathy. Jpn J Hum Genet 1997 42 187 91 9183998 10.1007/BF02766921

[12] Chinnery PF Howell N Andrews RM Turnbull DM Mitochondrial DNA analysis: polymorphisms and pathogenicity. J Med Genet 1999 36 505 10 10424809 PMC1734403

[13] Holt IJ Miller DH Harding AE Genetic heterogeneity and mitochondrial DNA heteroplasmy in Leber's hereditary optic neuropathy. J Med Genet 1989 26 739 43 2575667 10.1136/jmg.26.12.739 PMC1015752

[14] Chinnery PF Andrews RM Turnbull DM Howell NN Leber hereditary optic neuropathy: Does heteroplasmy influence the inheritance and expression of the G11778A mitochondrial DNA mutation? Am J Med Genet 2001 98 235 43 11169561 10.1002/1096-8628(20010122)98:3<235::aid-ajmg1086>3.0.co;2-o

[15] Brown MD Sun F Wallace DC Clustering of Caucasian Leber hereditary optic neuropathy patients containing the 11778 or 14484 mutations on an mtDNA lineage. Am J Hum Genet 1997 60 381 7 9012411 PMC1712415

[16] Hofmann S Jaksch M Bezold R Mertens S Aholt S Paprotta A Gerbitz KD Population genetics and disease susceptibility: characterization of central European haplogroups by mtDNA gene mutations, correlation with D loop variants and association with disease. Hum Mol Genet 1997 6 1835 46 9302261 10.1093/hmg/6.11.1835

[17] Lamminen T Huoponen K Sistonen P Juvonen V Lahermo P Aula P Nikoskelainen E Savontaus ML mtDNA haplotype analysis in Finnish families with leber hereditary optic neuroretinopathy. Eur J Hum Genet 1997 5 271 9 9412783

[18] Torroni A Petrozzi M D'Urbano L Sellitto D Zeviani M Carrara F Carducci C Leuzzi V Carelli V Barboni P De Negri A Scozzari R Haplotype and phylogenetic analyses suggest that one European-specific mtDNA background plays a role in the expression of Leber hereditary optic neuropathy by increasing the penetrance of the primary mutations 11778 and 14484. Am J Hum Genet 1997 60 1107 21 9150158 PMC1712418

[19] Carelli V Achilli A Valentino ML Rengo C Semino O Pala M Olivieri A Mattiazzi M Pallotti F Carrara F Zeviani M Leuzzi V Carducci C Valle G Simionati B Mendieta L Salomao S Belfort R Jr Sadun AA Torroni A Haplogroup effects and recombination of mitochondrial DNA: novel clues from the analysis of Leber hereditary optic neuropathy pedigrees. Am J Hum Genet 2006 78 564 74 16532388 10.1086/501236 PMC1424694

[20] Vilkki J Ott J Savontaus ML Aula P Nikoskelainen EK Optic atrophy in Leber hereditary optic neuroretinopathy is probably determined by an X-chromosomal gene closely linked to DXS7. Am J Hum Genet 1991 48 486 91 1998335 PMC1682980

[21] Ji Y Zhang AM Jia X Zhang YP Xiao X Li S Guo X Bandelt HJ Zhang Q Yao YG Mitochondrial DNA haplogroups M7b1'2 and M8a affect clinical expression of leber hereditary optic neuropathy in Chinese families with the m.11778G→a mutation. Am J Hum Genet 2008 83 760 8 19026397 10.1016/j.ajhg.2008.11.002 PMC2668067

[22] Ji Y Jia X Zhang Q Yao YG mtDNA haplogroup distribution in Chinese patients with Leber's hereditary optic neuropathy and G11778A mutation. Biochem Biophys Res Commun 2007 364 238 42 17942074 10.1016/j.bbrc.2007.09.111

[23] Bu XD Rotter JI X chromosome-linked and mitochondrial gene control of Leber hereditary optic neuropathy: evidence from segregation analysis for dependence on X chromosome inactivation. Proc Natl Acad Sci USA 1991 88 8198 202 1896469 10.1073/pnas.88.18.8198 PMC52474

[24] Nakamura M Fujiwara Y Yamamoto M The two locus control of Leber hereditary optic neuropathy and a high penetrance in Japanese pedigrees. Hum Genet 1993 91 339 41 8500789 10.1007/BF00217353

[25] Carvalho MR Muller B Rotzer E Berninger T Kommerell G Blankenagel A Savontaus ML Meitinger T Lorenz B Leber's hereditary optic neuroretinopathy and the X-chromosomal susceptibility factor: no linkage to DXs7. Hum Hered 1992 42 316 20 1360941 10.1159/000154089

[26] Chen JD Denton MJ X-chromosomal gene in Leber hereditary optic neuroretinopathy. Am J Hum Genet 1991 49 692 3 1882847 PMC1683133

[27] Juvonen V Vilkki J Aula P Nikoskelainen E Savontaus ML Reevaluation of the linkage of an optic atrophy susceptibility gene to X-chromosomal markers in Finnish families with Leber hereditary optic neuroretinopathy (LHON). Am J Hum Genet 1993 53 289 92 8317495 PMC1682246

[28] Hudson G Keers S Yu Wai Man P Griffiths P Huoponen K Savontaus ML Nikoskelainen E Zeviani M Carrara F Horvath R Karcagi V Spruijt L de Coo IF Smeets HJ Chinnery PF Identification of an X-chromosomal locus and haplotype modulating the phenotype of a mitochondrial DNA disorder. Am J Hum Genet 2005 77 1086 91 16380918 10.1086/498176 PMC1285165

[29] Shankar SP Fingert JH Carelli V Valentino ML King TM Daiger SP Salomao SR Berezovsky A Belfort R Jr Braun TA Sheffield VC Sadun AA Stone EM Evidence for a novel x–linked modifier locus for leber hereditary optic neuropathy. Ophthalmic Genet 2008 29 17 24 18363168 10.1080/13816810701867607

[30] Hirschhorn JN Lohmueller K Byrne E Hirschhorn K A comprehensive review of genetic association studies. Genet Med 2002 4 45 61 11882781 10.1097/00125817-200203000-00002

[31] Altmuller J Palmer LJ Fischer G Scherb H Wjst M Genomewide scans of complex human diseases: true linkage is hard to find. Am J Hum Genet 2001 69 936 50 11565063 10.1086/324069 PMC1274370

[32] Moonesinghe R Khoury MJ Janssens AC Most published research findings are false-but a little replication goes a long way. PLoS Med 2007 4 e28 17326704 10.1371/journal.pmed.0040028 PMC1808082

[33] Manly KF Reliability of statistical associations between genes and disease. Immunogenetics 2005 57 549 58 16086172 10.1007/s00251-005-0025-x

[34] McCarthy MI Abecasis GR Cardon LR Goldstein DB Little J Ioannidis JP Hirschhorn JN Genome-wide association studies for complex traits: consensus, uncertainty and challenges. Nat Rev Genet 2008 9 356 69 18398418 10.1038/nrg2344

[35] Wang P Li S Xiao X Jia X Jiao X Guo X Zhang Q High myopia is not associated with the SNPs in the TGIF, lumican, TGFB1, and HGF genes. Invest Ophthalmol Vis Sci 2009 50 1546 51 19060265 10.1167/iovs.08-2537

[36] Newman NJ From genotype to phenotype in Leber hereditary optic neuropathy: still more questions than answers. J Neuroophthalmol 2002 22 257 61 12464728 10.1097/00041327-200212000-00001

[37] Marotta R Chin J Quigley A Katsabanis S Kapsa R Byrne E Collins S Diagnostic screening of mitochondrial DNA mutations in Australian adults 1990–2001. Intern Med J 2004 34 10 9 14748908 10.1111/j.1444-0903.2004.t01-3-.x

[38] Pegoraro E Vettori A Valentino ML Molon A Mostacciuolo ML Howell N Carelli V X-inactivation pattern in multiple tissues from two Leber's hereditary optic neuropathy (LHON) patients. Am J Med Genet A 2003 119A 37 40 12707956 10.1002/ajmg.a.10211

[39] Barkley NA Dean RE Pittman RN Wang ML Holbrook CC Pederson GA Genetic diversity of cultivated and wild-type peanuts evaluated with M13-tailed SSR markers and sequencing. Genet Res 2007 89 93 106 17669229 10.1017/S0016672307008695

[40] Nakanishi H Yamada R Gotoh N Hayashi H Yamashiro K Shimada N Ohno-Matsui K Mochizuki M Saito M Iida T Matsuo K Tajima K Yoshimura N Matsuda F A genome-wide association analysis identified a novel susceptible locus for pathological myopia at 11q24.1. PLoS Genet 2009 5 e1000660 19779542 10.1371/journal.pgen.1000660 PMC2735651

